# Grandchild contact and grandparents’ positive and negative emotions in daily life: an analysis using self-report and language use data

**DOI:** 10.1093/geronb/gbag043

**Published:** 2026-03-21

**Authors:** Flavia S Chereches, Yvonne Brehmer, Gabriel Olaru, Nicola Ballhausen

**Affiliations:** Department of Developmental Psychology, Tilburg University, Tilburg, The Netherlands; Department of Developmental Psychology, Tilburg University, Tilburg, The Netherlands; Aging Research Center, Karolinska Institutet, Stockholm, Sweden; Department of Differential Psychology and Psychological Assessment, Charlotte Fresenius Hochschule, Cologne, Germany; Department of Developmental Psychology, Tilburg University, Tilburg, The Netherlands; Centre for the Interdisciplinary Study of Gerontology and Vulnerability, University of Geneva, Geneva, Switzerland; (Psychological Sciences Section)

**Keywords:** Grandparenthood, EAR, Affective well-being, Social interactions

## Abstract

**Objectives:**

Relationships between grandparents and grandchildren are a vital aspect of family life, yet little is known about how grandchild contact affects grandparents’ emotions in daily life. This study aims to examine the short-term impact of grandchild contact on grandparents’ positive and negative emotions.

**Methods:**

Analyzing a sample of 125 grandparents from the “Daily Experiences and Wellbeing study” followed for a period of 5 days (6 assessments per day) with multilevel structural equation modeling, we compared grandparents’ positive and negative emotions when being with their grandchildren to contact with close others and to time with their spouse or alone. Additionally, using snippets of ambient sound recordings, we investigated whether grandparents used more positive and fewer negative emotion-related words when they had contact with grandchildren compared to time spent with others.

**Results:**

Interactions with grandchildren were associated with increased positive emotions, especially more love and pride, and a higher use of positive words compared to interactions with other close social members and time spent alone or with a spouse. We found limited evidence that grandparents also reported feeling sadder and more worried when with their grandchildren, although these effects were less robust.

**Discussion:**

Our findings, based on multiple assessment methods (self-report and language use), indicate that grandchild contact is associated with increases in specific self-reported emotions, which are also mirrored in the language used in daily life. These particular emotions (love and pride) may highlight the unique relationship between grandchildren and grandparents, marked by affection and opportunities to express generativity.

Grandparenthood is considered one of the most important roles of middle and late adulthood (Reitzes & Mutran, 2002). The significance of this role is well captured by Erikson’s theory of psychosocial development, particularly the stage of *generativity versus stagnation*, in which individuals seek to contribute to the well-being of future generations (Erikson, 1963). Contact with grandchildren is often seen as a tangible way to express generativity, enabling older adults to pass on values and maintain a sense of legacy (see also the introduction of grand-generativity in Erikson’s later work; Erikson et al., 1994). Moreover, role theory indicates that holding socially valued roles enhances well-being (Adelmann, 1994; Biddle, 1986; Sieber, 1974). In the context of grandparenthood, grandchildren may provide opportunities for meaningful interactions and social recognition, which could support grandparents’ well-being (Dunifon et al., 2020; Villar et al., 2012).

However, research investigating the effect of contact with grandchildren (most often studied as grandchild care) and grandparents’ well-being has been mixed so far. Some research found positive (Moore & Rosenthal, 2015; Tanskanen et al., 2019), null (Herrera et al., 2022; Krämer et al., 2023), and even negative effects of grandchild interaction (through caregiving or contact) on grandparents’ well-being (Quirke et al., 2021). Because much of this work has focused on care provided to grandchildren, it implicitly assumes that grandparents are the primary initiators of contact. Yet, as grandchildren grow older, they can also provide companionship or functional assistance to grandparents. In this context, contact with grandchildren is also expected to be linked with grandparents’ well-being. For instance, Moorman and Stokes (2016), in a U.S. sample, found that receiving functional support from grandchildren without providing support in return was associated with poorer grandparent well-being.

Past research on grandparenthood and well-being has most often compared grandparents who differ in the extent of their involvement with grandchildren (including grandparents vs. non-grandparents), or in other individual or grandparenthood-related characteristics (for a review, see Danielsbacka et al. [2022]). However, despite the relevance of such between-person comparisons, these paradigms make it difficult to determine whether greater well-being is truly a consequence of grandchild contact/care or instead reflects preexisting differences. For instance, grandparents who are healthier and cognitively fitter could both be more likely to engage in caregiving and initiate contact (Chereches et al., 2025) and could report higher well-being (Glaser & Di Gessa, 2025). To understand the unique contribution of grandchild contact to grandparents’ well-being, it is therefore necessary to examine these dynamics within individuals; i.e., to compare grandparents’ well-being in moments when they are having contact with grandchildren to moments when they are not. Intensive longitudinal designs, which assess the same participants repeatedly (multiple times per day and/or across multiple days), offer a way to capture short-term fluctuations in well-being that may unfold around the fluctuation of moments of contact. However, such fine-grained within-person designs remain scarce in the grandparenthood literature.

## What are the short-term effects of grandchild contact on grandparents’ well-being?

Limited past research has examined the effects of contact with grandchildren on grandparents’ momentary well-being. A notable exception is the study of Dunifon et al. (2020), who examined the within-person effects of grandchild contact on co-residing grandparents’ well-being using a day reconstruction method in a U.S. sample. Comparing time spent with grandchildren to that spent with other social contacts and alone, the authors showed that grandparents experienced greater happiness and meaningfulness when with their grandchildren compared to other times. Time spent with grandchildren was also associated with less sadness than time spent alone, but not compared to time spent with other social contacts. Stress and tiredness, the other two emotions investigated, remained unaffected by grandchild contact. These results highlight short-term emotional benefits of contact with grandchildren, with some benefits unique to contact with grandchildren (happiness and meaningfulness), and others similar to those of other social contacts (e.g., sadness diminished only when compared to time spent alone). Moreover, as also observed in past longitudinal studies (e.g., Condon et al., 2018), grandchild contact does not seem to influence all emotions equally, suggesting the necessity to investigate specific emotions beyond broader well-being constructs.

## Overlooked emotions in the context of grandparenthood: which emotions are specific to grandchild contact?

Although Dunifon et al. (2020)’s study is one of the few to examine grandchild contact over a shorter period using specific emotions (rather than broad positive/negative categories), it did not include some emotions that could be particularly relevant to grandchild contact. Emotions such as pride, love, and worry might be closely linked to interactions with grandchildren, considering their unique role as close kin and the high emotional investment grandparents generally have in the relationship (Duflos et al., 2022; Gattai & Musatti, 1999; Mansson, 2016). Engaging with grandchildren allows for generative actions, or the passing on of one’s skills and knowledge to future generations (Erikson, 1963; Villar et al., 2012) while reflecting the continuity of the self across generations (Celdrán, 2024). In line with the intergenerational stake hypothesis (Bengtson & Kuypers, 1971) grandparents may view their grandchildren as a continuation of their lives and experience increased affection toward their grandchildren (Giarrusso et al., 2001). Qualitative studies confirm this perspective – grandparents seem to experience a strong sense of ownership and pride for their grandchildren’s achievements (Mansson, 2016), as well as feelings of love and fulfillment (Kemp, 2005). 

In contrast, emotions such as contentment or loneliness may also be elicited in other social contexts and may not be uniquely linked to grandparent–grandchild interactions. However, in the absence of empirical evidence, it remains possible that grandchild contact also shapes these emotions. Therefore, in this study, we investigate which *specific* positive emotions (e.g., proud, content, loved, calm, energized) and negative emotions (e.g., nervous/worried, irritated, bored, lonely, sad) are affected by grandchild contact.

## Spoken emotion-related words: indicators for how grandparents feel?

The electronically activated recorder method (EAR; Mehl et al., 2001) is an increasingly used non-intrusive method for understanding everyday behavior and language use, and it has also been successfully deployed in older adults (Haas et al., 2022). By collecting ambient sound and speech, EAR enables general language analysis and investigations of the content of words individuals use. The emotiveness of communicated words has been linked to individuals’ well-being (Vine et al., 2020). For example, estimates of positive and negative emotions from EAR were associated with participants’ reported emotional valence during ecological momentary assessments (EMA), both within- and between-person (Carlier et al., 2022). Research among older adults found that higher expression of positive emotion-related words was linked to momentary positive but not negative emotions (Zhang et al., 2025). Despite some limitations (e.g., contexts demanding positive words without accompanying positive emotions; Fast & Funder, 2008), language analysis of conversational speech seems a promising tool for objectively capturing momentary emotions (Munin et al., 2025).

Within grandparenthood research, studying whether grandparents use more positively valenced words when with grandchildren than with others could provide more objective insights into their behavior and emotions in their interactions, beyond self-reported measures. If the grandparent role involves nurturing and transmitting values to future generations, as expressed in theories (Celdrán, 2024; Erikson, 1963; Erikson et al., 1994), then one would expect this to be reflected in grandparents’ language through increased positively valenced words and decreased negatively valenced words. This also aligns with communication accommodation theory (Giles, 1973), which posits that individuals adjust their communication to create or reduce distance from their social partners. In this case, grandparents may adjust their language to connect with or support their grandchildren. Moreover, it could be informative to study whether grandparents’ perceived emotions align with the emotiveness of their language.

## Present study

This study used EMA to examine associations between grandchild contact and grandparents’ affective well-being, as captured via momentary self-reports and recordings of their spoken words. We compared interactions with grandchildren to moments without grandchild interactions and with interactions with other individuals (moments in which there was social contact with close social members but not grandchildren, even if nonsocial members were present alongside close ones). This was done to determine whether observed effects were unique to grandchild contact. Additionally, we compared grandchild contact to time spent alone or with a spouse to capture instances of limited social engagement. We categorized time spent with a spouse alongside time spent alone, as there were few instances and few participants who were alone during the study period (see also Birditt et al., 2019). We considered this appropriate because for married and cohabiting participants, interactions with their spouse may represent the “standard” (Ng et al., 2022).

Building on past research (Dunifon et al., 2020), we preregistered the following hypotheses:

*Hypothesis 1:* Grandparents reported more positive emotions and less negative emotions when they were in contact with their grandchildren compared to (a) when they interacted with other individuals and (b) when they were alone or just with their spouse.

*Hypothesis 2:* Grandparents used more positive and fewer negative words during times in which they had contact with their grandchildren compared to (a) when they interacted with other individuals and (b) were alone or just with their spouse.

Finally, although not preregistered, in addition to the within-person effects, allowing us to see how day-to-day fluctuations in grandchild contact relate to emotions within the same person, we also report between-person associations. These would inform us if individuals who had more contact with grandchildren during the study period differ in their average positive and negative emotions from individuals having less contact with grandchildren.

## Method

### Participants

This preregistered study (https://osf.io/9w5fx/?view_only=69f80f8832b34c16abe2e1dacbf48bbd) used data from the publicly available Daily Experiences and Wellbeing in Late Life (DEWS; Fingerman et al., 2022). DEWS originally included 333 adults aged 65 and older who reside at home and do not work full-time from Austin, TX (83% of the participants were White). They completed a baseline assessment and conducted intensive longitudinal data collection for a period of 5–6 days, with data collected every 3 hr, resulting in approximately six surveys per day based on participants’ self-reported wake-up and bedtimes, achieving a compliance rate of 71% (Zhou et al., 2024). This data collection included assessments of daily social encounters, emotions, and EAR data (see Fingerman et al., 2022, for a complete description of the study).

Only participants who had at least one contact with grandchildren during the study period were selected, resulting in a sample of 125 grandparents (2,644 observations). Participants were on average approximately 75 years (age was reported in 3-year increments; see Supplementary material for details). Most participants (56%) were at least college graduates, 70.63% were women, and 55.2% were married or cohabiting with a partner, and 83.47% were retired or not working, 22.4% of participants reported that they provide regular care to someone (e.g., spouse, parent, grandchild), with only 11.2% reporting to provide care to grandchildren.

On average, participants completed 20.78 surveys (*SD *= 5.13) and reported on average five contact moments with their grandchildren. The average age of the youngest grandchild with whom contact occurred was around 15 years, with only 36% of the sample reporting contact with grandchildren below 10 years of age.

### Measures

#### Contact with grandchildren

Participants completed a baseline assessment where they listed their closest social contacts. In subsequent daily assessments, they reported interactions within the past 3 hr, specifying whether they engaged with the first 10 listed closest social contacts or other individuals. Grandchild contact included any face-to-face or remote interactions, even when others were present, since fewer than 100 instances involved exclusive grandchild interactions. To increase the number of observations and because prior research has shown that well-being benefits from remote contact with grandchildren (Alonso Ruiz et al., 2022; Wei et al., 2023), and remote and face-to-face contact are highly correlated (Chereches et al., 2025), both types of contact were considered grandchild contact.

#### Positive and negative emotions

Participants rated several presented emotions over the past 3 hr on a 1–5 scale (1 = *not at all*, 2 = *a little*, 3 = *somewhat*, 4 = *quite a bit*, and 5 = *a great deal*). Positive emotions included contentment, love, pride, calm, and energy. Negative emotions included boredom, loneliness, sadness, irritation, and nervousness/worry. We analyzed each emotion separately and as averaged positive and negative emotion indices (Negative emotions: McDonald’s *ω*_between_ = .92, *ω*_within_ = .59; Positive emotions: McDonald’s *ω*_between_ = .76, *ω*_within_ = .50).

#### EAR data

Ambient sounds and participants’ conversations were recorded using the EAR for 30 s every 7 min during waking hours. As reported elsewhere (Zhang et al., 2023), the EAR generated 147,990 sound files, out of which 67,572 had audible sound (e.g., conversations, environmental sound). The speech was transcribed verbatim by trained research assistants of the original project, with the social partners’ speech not being transcribed due to ethical concerns. Transcripts were analyzed with Linguistic Inquiry and Word Count 2015 (LIWC; Pennebaker et al., 2015), a widely used tool for inferring emotional states from verbal behavior by comparing the text to its embedded dictionary. Examples of positive words from the LIWC dictionary include “love” and “sweet,” and for negative words “hurt” and “ugly.” For each transcribed snippet, positive and negative emotion scores were calculated as the percentage of positively or negatively valenced words. Intervals without speech were scored as missing.

In the publicly available dataset, these scores were already averaged into 3-hr periods to align with the self-report assessments. There were fewer EAR observations than self-report data, as participants could delete private recordings or the research team removed them during transcription (for the subsample of grandparents, 63% of all observations contained EAR data). EAR recordings may include speech from diverse contexts, such as interactions with others, background conversations, or self-talk, within the same 3-hr window that involved grandchild contact. Hence, rather than capturing only linguistic responses directed toward grandchildren, these recordings likely reflect a broader “emotional override,” or a more general shift in emotional tone associated with periods that included grandchild contact.

### Statistical analyses

To test our hypotheses, we used multilevel structural equation modeling (MSEM) in Mplus Version 8.10 (Muthén & Muthén, 2017), using the Bayes estimator and default diffuse priors in all models (Asparouhov & Muthén, 2010 in Supplementary Material). We worked with two-level models (observations nested in participants) and used latent centering for our Level 1 predictors, as recommended given our binary predictor (i.e., grandchild contact 1/0; Asparouhov & Muthén, 2019). Predictors were thus split in-between- and within-person latent components, where the between-person components are similar to person means corrected for measurement error and the within-person components are time-specific deviations from these latent intercepts. We expected the effects to differ between individuals, hence we used random slopes for the focal predictors (except for models comparing grandchild contact to time alone, as there were fewer observations). To make inferences, we used Bayesian credible intervals. Models were considered converged if the Potential Scale Reduction criterion was close to 1.01 (Brooks & Gelman, 1998 in Supplementary Material). Note that we controlled for age (see Supplementary material for age group variable), gender (0 = *woman*, 1 = *man*), and marital status (0 = *not married*, 1 = *married or cohabiting with a partner*). We modeled Level 1 and Level 2 effects simultaneously, as permitted by the MSEM framework. For the models investigating speech data, we included the number of words as an additional predictor both at the within- and between-level. For the between-person results and the full model specification in formula form, see Supplementary material.

We ran the models for each emotion separately, under three increasingly stringent conditions: We compared grandparents’ affective well-being during moments of grandchild contact (667 observations) to (a) all other occasions (1,977 observations, including time alone and contact with anyone else), (b) contact with other close social network members identified at baseline (1,472 observations), and (c) time spent alone or with the spouse/partner (563 observations). Contact with grandchildren was always coded as 1 (with 0 representing the other conditions).

## Results

### Descriptives

Grandparents reported contact with grandchildren in 25.24% of assessments, with an average of 5.34 grandchild contact moments per participant (range 1 to 25). Participants experienced moderate to high levels of positive emotions and low levels of negative emotions (see Table 1). The variables with the strongest within-person fluctuations were the valence of spoken words (positive/negative words) and certain negative emotions (sad, worried, and lonely). In contrast, positive affect was more stable over time, particularly the emotions of pride and love.

**Table 1:** Descriptive statistics and ICC for variables of the whole sample.

Variable	*M*	*SD*	ICC
**Energetic**	3.07	1.05	.46
**Content**	3.83	0.95	.55
**Loved**	4.05	1.06	.71
**Proud**	2.60	1.36	.76
**Calm**	3.97	0.86	.56
**Bored**	1.13	0.43	.62
**Lonely**	1.10	0.38	.32
**Sad**	1.17	0.47	.31
**Irritated**	1.22	0.56	.31
**Nervous**	1.23	0.54	.42
**Positive emotions**	3.50	0.73	.72
**Negative emotions**	1.17	0.34	.56
**EAR positive words**	2.04	2.57	.18
**EAR negative words**	0.28	0.64	.11
**Pleasantness**	4.70	0.71	.31

*Note.* Positive emotions = averaged across all specific positive emotions; negative emotions = averaged across all specific negative emotions; EAR positive words = percentage of spoken positive emotion-related words; EAR negative words = percentage of spoken negative emotion-related words; pleasantness = average pleasantness of all social interactions; ICC = intraclass correlation coefficient, denoting the proportion of variance at the between-person level. By whole sample, we mean moments of grandchild contact and moments when grandchildren were absent (i.e., when grandparents were alone, with their spouse, or with other social members).

As shown in Table 2, at the within-person level, we found that grandchild contact (compared to no grandchild contact) was associated with slightly higher positive emotions and increased use of positive words, but not with increased negative emotions. The correlation between word use and emotions was rather weak for positive emotions and nonsignificant for negative emotions.

**Table 2:** Within- and between-person correlations of grandchild contact and emotions.

Variable	Grandchild contact	Positive emotions	Negative emotions	EAR positive words	EAR negative words	Pleasantness
**Within-person**						
Contact		.09***	.02	.13***	.03	.06**
Energetic	.03	.61***	−.12***	.07***	−.02	−.03
Content	.04*	.67***	−.29***	.06*	.02	.13***
Loved	.10***	.54***	−.09***	.01	.05	.07*
Proud	.09***	.59***	−.09***	.10***	.01	.07*
Calm	−.01	.53***	−.32***	−.01	−.01	.12***
Bored	−.02	−.15***	.44***	.00	−.01	−.08**
Lonely	.02	−.17***	.56***	−.02	−.05*	−.05
Sad	.03	−.15***	.66***	.02	−.01	−.24***
Irritated	.00	−.25***	.67***	−.02	.01	−.19***
Nervous	.04	−.19***	.67***	.00	−.01	−18***
Pleasantness	.06**	.11***	−.29***	.03	.00	
**Between−person**						
Contact		−.26**	.29**	−.15	.34**	−.16
Energetic	−.10	.56***	−.23**	.10	.03	.34***
Content	−.34***	.78***	−.54***	.03	−.16	.42***
Loved	−.23*	.79***	−.25**	.05	−.03	.39***
Proud	−.05	.72***	−.09	−.05	.11	.24*
Calm	−.28**	.78***	−.49***	−.03**	−.27**	.31***
Bored	.37***	−.32***	.79***	−.16	.63***	−.30**
Lonely	.17	−.34***	.83***	−.13	.31*	−.25*
Sad	.19*	−.32***	.82***	.00	.40**	−.39**
Irritated	.20*	−.31***	.78***	.04	.73***	−.43***
Nervous	.18*	−.34***	.90***	−.06	.34**	−.30**
Pleasantness	−.18*	.29***	−.26**	.08	−.04	

*Note*. Contact = grandchild contact (versus *all* other occasions); positive emotions = averaged across all specific positive emotions; negative emotions = averaged across all specific negative emotions; EAR positive emotions = spoken positive emotion-related words; EAR negative = spoken negative emotion-related words; pleasantness = average pleasantness of all social interactions. These reflect bivariate correlations, within-person and between-person, run in the frequentist framework.

*p < .05.

**p < .01.

***p < .005.

At the between-person level, the pattern contrasted with what we observed within-persons: Grandparents who had more contact with their grandchildren reported experiencing more negative and less positive emotions, and using more negative words than those with less contact. Furthermore, negative emotions and word use were moderately correlated between persons, but we found no link for positive emotions.

Checking between-person associations with baseline information, we found that grandparents who met their grandchildren more often during the study period were less educated (*r *= −.36), had more grandchildren (*r* = .20), older grandchildren (*r* = .22), and reported worse subjective health (*r *= −.25), and more depressive symptoms (*r* = .26) than those who had less contact with their grandchildren (all *p*_s_ < .05; Table S3). For between-person effects of multilevel models, see Supplementary material.

### Grandchild contact and grandparents’ emotions

#### Self-reported emotions

As shown in Table 3, when grandparents had contact with their grandchildren, they experienced more positive affect, particularly feeling loved and proud, than in all other conditions (i.e., contact with others and time alone or just with their spouse). These effects were strongest when compared to being alone or with their spouse (positive affect *β* = .16) and weakest when compared to contact with other close people (positive affect *β* = .06). Grandparents also reported feeling more energetic while interacting with their grandchildren, but only in comparison to being alone or with their spouse.

**Table 3:** Within-person findings: unstandardized and (standardized) estimates comparing grandchild contact to all other conditions.

Variable	Grandchildren not present		Contact with other social members		Time alone or just with spouse	
	Estimate	95% CI	Estimate	95% CI	Estimate	95% CI
**Energetic**	0.066 (.032)	[−0.017, 0.154]	0.025 (.011)	[−0.065, 0.115]	**0.246 (.138)**	**[0.140, 0.352]**
**Content**	0.056 (.032)	[−0.040, 0.149]	0.069 (.038)	[−0.031, 0.165]	0.058 (.036)	[−0.040, 0.153]
**Loved**	**0.142 (.092)**	**[0.064, 0.220]**	**0.077 (.052)**	**[0.010, 0.143]**	**0.214 (.140)**	**[0.124, 0.307]**
**Proud**	**0.151 (.083)**	**[0.059, 0.239]**	**0.119 (.067)**	**[0.017, 0.216]**	**0.299 (.183)**	**[0.203, 0.394]**
**Calm**	−0.020 (−.011)	[−0.092, 0.055]	−0.002 (−.004)	[−0.080, 0.075]	−0.078 (−.052)	[−0.167, 0.013]
**Bored**	0.021 (.016)	[−0.030, 0.074]	0.023 (.007)	[−0.040, 0.090]	0.020 (.028)	[−0.022, 0.062]
**Lonely**	0.029 (.036)	[−0.015, 0.075]	0.022 (.029)	[−0.021, 0.067]	−0.004 (−.004)	[−0.056, 0.050]
**Sad**	**0.083 (.063)**	**[0.005, 0.163]**	0.070 (.054)	[−0.013, 0.153]	0.039 (.038)	[−0.022, 0.100]
**Irritated**	0.002 (.001)	[−0.052, 0.057]	−0.010 (−.009)	[−0.071, 0.052]	0.026 (.023)	[−0.040, 0.093]
**Nervous/worried**	**0.064 (.055)**	**[0.004, 0.129]**	0.040 (.035)	[−0.024, 0.107]	**0.075 (.079)**	**[0.019, 0.131]**
**Negative emotions**	**0.047 (.062)**	**[0.004, 0.093]**	0.033 (.043)	[−0.011, 0.081]	0.032 (.053)	[−0.004, 0.066]
**Positive emotions**	**0.087 (.084)**	**[0.037, 0.136]**	**0.065 (.060)**	**[0.011, 0.116]**	**0.152 (.160)**	**[0.097, 0.208]**
**EAR negative emotions**	0.014 (.013)	[−0.067, 0.095]	−0.018 (.005)	[−0.106, 0.082]	0.013 (.009)	[−0.105, 0.132]
**EAR positive emotions**	**0.530 (.084)**	**[0.180, 0.871]**	**0.555 (.093)**	**[0.185, 0.913]**	**0.980 (.182)**	**[0.563, 1.398]**

*Note.* Grandchildren not present = results on the full sample, comparing situations of grandchild contact with interacting with other social members or time spent alone or with the spouse only; contact with other social members = results comparing situations of grandchild contact with datapoints in which social interactions occurred (i.e., spouse or other social members); time alone or just with spouse = results comparing situations of grandchild contact with datapoints where there was no social contact, or just contact with spouse; EAR negative = spoken negative emotion-related words; EAR positive emotions = spoken positive emotion-related words. Values represent unstandardized estimates, and standardized estimates in parentheses. In the squared brackets, we present 95% credible intervals (CIs). Coefficients in bold indicate effects for which the credibility interval does not include 0. Note that for models comparing grandchild contact to absence of contact, we run models without a random slope, to facilitate convergence given the lower number of observations.

Grandparents also experienced more negative affect when interacting with their grandchildren, specifically feeling sadder and more nervous or worried, as compared to no grandchild contact. Although the effect sizes were similar in all three comparisons (e.g., negative affect *β*s = .05 to .06), credibility intervals were larger in the more restrictive comparisons (e.g., time alone or with spouse), possibly because of the lower number of occasions available for estimation.

#### Spoken words

Analyzing the EAR data, we found that grandparents used more positively valenced words when they had contact with their grandchildren compared to all other conditions. No effects were observed for the negative emotion related words.

#### Additional analyses

##### Accounting for carry-over effects and time

As prior emotions may linger and influence subsequent emotion levels, we included an autoregressive effect for the emotion (not preregistered; Koval et al., 2015) and added time in the study as an additional predictor in the models. These analyses were limited to the dataset with all available datapoints (i.e., grandchild contact vs. no contact), as assessments occurred in a consistent sequence and at regular intervals (unlike other comparisons, based on subsets of data, and consequently with the previous time point possibly missing). We observed standardized autoregressive effects ranging from *β* = .04 to *β* = .23 across emotions. There was no effect of time (except for a small increase in pride and decrease in calmness, Table S4). Although the explained variance increased once including autoregressive effects in the model, the effects on pride, love, and average positive emotions were still present, and comparable in their size to the previous model. Effects of grandchild contact on sadness, nervousness/worries, and overall negative emotions were no longer observed (Tables S5 and S6). These findings suggest that the earlier associations between grandchild contact and negative emotions may have been driven by other factors that continued to influence grandparents’ momentary emotional states.

##### Accounting for perceived pain

Because both grandparents and grandchildren are older (grandparents, on average, more than 70 years old, with their youngest grandchild on average around 15 years of age), contact may have occurred in response to grandparents’ discomfort or health issues. To account for this potential confound, we added participants’ reported pain (0 = *no pain*, 3 = *severe pain*) within the past 3 hr as a within-person covariate. The explained within-person variance was similar to models without this covariate, and the results remained unchanged (Tables S11–S13).

##### Accounting for pleasantness

To determine whether the within-person effects were influenced by the pleasantness of social interactions, we first ran dependent *t*-tests within the frequentist framework (where pleasantness was rated for each social partner, on a 5-point scale, from 1 = *unpleasant* to 5 = *pleasant*). We found that contact with grandchildren was on average rated as more pleasant than interactions with other close individuals (*d *= 0.19, *p* = .039), but not compared to interactions with their spouse (*p* = .318; see Table S7 for detailed results). Subsequently, we added the pleasantness of the social interactions as a within-person covariate in our multilevel models comparing grandchild contact to contact with other close social members. Note that when multiple interactions occurred within one assessment (e.g., interactions with three close individuals in the past 3 hr, resulting in three pleasantness reports), we used the average of these reports as a within-person covariate. The models controlling for average momentary pleasantness of the social interactions at Level 1 showed that all previously observed effects remained (and there was also no effect of contact with grandchildren on sadness, worry, and negative emotions). The explained within-person variance was mostly similar to models without this covariate (Tables S8 and S9). Pleasantness of social interaction had effects on emotions in the expected direction; it increased positive emotions and decreased negative emotions (Table S10). Thus, the effect of grandchild contact on positive emotions (pride, love) and positive emotion words did not appear to be driven by the pleasantness of the social interaction.

## Discussion

This research investigated the short-term effects of grandchild contact on grandparents’ positive and negative emotions compared to contact with other close social members and being alone or with their spouse. Within-person, we found that grandchild contact was linked to increased use of positively valenced words, higher overall positive affect, especially pride and love, as well as increased negative affect, particularly worry and sadness.

### How do grandparents experience contact with grandchildren?

Our within-person findings align, at least partly, with Dunifon et al. (2020), who reported that grandparents experience more positive emotions when spending time with their grandchildren compared to other social contacts or being alone. However, we extended their findings to specific emotions that were less studied in quantitative research, such as pride and love. The increase in pride observed in this sample appeared to be specific to interactions with grandchildren, holding even when compared to contact with close other individuals, members of one’s close social network. Our findings thus extend theoretical work suggesting that grandparents derive pride from their grandparent role (Mansson, 2016). The presence of grandchildren possibly strengthens older adults’ role as mentors, enabling them to express generativity (Celdrán, 2024) and reaffirm their role within the family (Kemp, 2005).

Grandparents also felt more loved when with their grandchildren than when they were alone or had contact with other social members. This echoes past qualitative research indicating that interacting with grandchildren offers a unique emotional experience, with Italian grandparents describing their feelings toward their grandchildren as “a new feeling” or a “different kind of love” (Gattai & Musatti, 1999). Additionally, our findings regarding love and pride align with Mansson’s (2016) thematic analysis on a U.S. sample, which identified mutual affection (the giving and receiving of love), shared activities with grandchildren, and pride in watching their grandchildren as the three most common themes that grandparents find rewarding in their relationships.

Contact with grandchildren was also linked to increased feelings of energy (a positive, high-arousal emotion) compared to being alone or with a spouse. Being younger, grandchildren may surpass their grandparents in energy levels, potentially stimulating them (Caputo et al., 2024). However, this might not be exclusive to grandchildren; energy levels were similar when in contact with other close individuals.

Contact with grandchildren appeared to also be linked to negative emotions. Grandparents felt more nervous/worried, and sad when reporting interactions with their grandchildren than when they were not in contact. Although the source of these emotions is unclear, past research suggests that grandparents worry about their grandchildren’s safety and well-being (Fingerman, 1998). Additionally, grandchildren may also serve as a reminder of aging, the transience of life, and the loss of abilities (Bordone & Arpino, 2016). They might evoke bittersweet and mixed feelings: a sense of pride in watching one’s grandchild, reflecting the continuity of oneself, but also nostalgia and sadness at the passing of time and life’s finiteness (Ersner-Hershfield et al., 2008). This might also be a result of the sample investigated, as participants were, on average, more than 70 years old, with their youngest grandchild on average around 15 years of age. It is also possible that these effects vary across subgroups of grandparents. For instance, the link between grandchild contact and sadness might be stronger among those in poorer health, for whom such interactions could more vividly evoke feelings of finiteness. Although this data did not allow us to formally test such moderation effects, future research should examine whether individual characteristics, such as health, shape these emotional responses.

However, it should be highlighted that, unlike the consistent effects observed for positively valenced emotions across all comparison conditions and sensitivity analyses, the effects for negative emotions appeared only in some comparisons. They also vanished when controlling for the emotion at the previous assessment. This suggests that the negative emotions might be less specific to grandchild contact but could be caused by events that occurred prior to the contact moment and still linger. Moreover, the credibility intervals were often near zero, indicating uncertainty in the estimates. Yet, past research on Dutch grandparents suggests that grandchild contact may imply a coexistence of positive and negative experiences (Grünwald et al., 2024), and hence future research should aim to replicate these findings while also accounting for the possibility of mixed emotions (Barford et al., 2020).

We found no within-person effect of grandchild contact on grandparents’ boredom, loneliness, or irritation, even when compared to being alone or with a spouse. Although in this sample participants were rarely alone, in these moments, they might have been comfortable, given research suggesting benefits of solitude (i.e., lower negative emotions; Birditt et al., 2019). There was also no effect of grandchild contact on irritation, contentment, or calmness, which might not be specific to (grandchild) contact but caused by other influencing factors (e.g., other social contacts or events) occurring throughout the day.

### Different lenses on grandchild contact: spoken words

Grandparenthood entails societal expectations and norms, including expressing love and supporting grandchildren (Mansilla-Domínguez et al., 2024). Consequently, when asked about their experiences, grandparents may offer socially desirable responses instead of completely authentic accounts (see Chereches et al., 2025, for a discussion on the challenges of evaluating grandparents’ perceptions of grandparenthood). To obtain alternative insights into individuals’ behavior and emotions, we complemented self-reported emotional data with EAR (speech) data.

Our analyses indicated that when contact with grandchildren was reported in the past 3 hr, grandparents also used more positively valenced words than when interacting with other social members or being alone. This may (at least partly) reflect the positive emotions grandparents experience during grandchild contact, supporting the obtained self-report findings. Using more positively valenced words might also reflect grandparents’ conscious effort to give grandchildren a good time, aligning with qualitative research that shows grandparents adjust their behavior to enhance their grandchildren’s health and well-being (Chambers et al., 2022) and the idea that grandparenthood is seen as a key opportunity for generativity (Celdrán, 2024). However, because the language samples may also include interactions with others that occurred within the same 3-hr window, the observed results could also reflect the carry-over or lingering influence of emotions from contact with grandchildren that still shape subsequent behavior or speech (even when the grandchildren are not present). Future research should replicate these findings while capturing more precisely the timing of grandchild contact and its aftermath, and exploring beyond emotional word use to include conversation topics and nonverbal emotional cues. For example, sighing, an observable indicator of depression (Robbins et al., 2011), may signal negative emotions, whereas laughter could serve as a behavioral marker for positive emotions (Ramírez-Esparza et al., 2019).

Nevertheless, grandparents perceived contact with grandchildren as more pleasant than contact with other close social members. Furthermore, in our follow-up sensitivity analysis, controlling for the within-person effects of interaction pleasantness did not eliminate the previously observed effects. This suggests that contact with grandchildren is experienced with changes in (spoken) emotions, irrespective of the pleasantness of the social interactions.

### Between-person findings: different pattern of results?

Between-person results contrasted with the within-person findings. Grandparents who spent more time with their grandchildren during the study period experienced, on average, more negative and fewer positive emotions, aligning with research that links greater grandchild contact to lower well-being for grandparents in the United States (Moorman & Stokes, 2016). However, grandparents with more grandchild contact were, in general, less educated, had older grandchildren, and had more grandchildren. As such, they might have been of lower socioeconomic status than those grandparents with less contact, which could explain their lower physical and mental health, and consequently lower affect. Although the negative association between grandparents’ health and grandchild contact contrasts with previous studies that found healthier European grandparents engage more with grandchildren than those who are less healthy (Di Gessa et al., 2016), this relationship is more applicable to a caregiving context. Most of the grandchildren in the current study were generally old enough to initiate contact with their grandparents themselves, and grandparents of older grandchildren also reported overall more contact. This may be because grandparents seek their grandchildren’s support, or because grandchildren increase contact with grandparents in response to grandparents’ frailty (as potentially indicated by the lower subjective health and higher levels of depression in grandparents having frequent contact with their grandchildren). Yet, receiving help from grandchildren challenges the typical pattern of downward support (from the older to the younger generations; Kemp, 2005), which could further compromise grandparents’ mental health (Moorman & Stokes, 2016).

However, these (between-person) higher levels of negative affect do not imply that in the short term, grandparents cannot enjoy the company of their grandchildren, which might be experienced as particularly uplifting and as an opportunity to express their worries and sadness (see within-person findings). Nonetheless, the differences between the between-person and within-person results underline that these levels of analysis do not always align (Chereches et al., 2023), emphasizing the need to consider both in future research.

### Strengths and limitations

This study examines how contact with grandchildren affects grandparents’ emotions, combining self-reports of emotions that were less studied in grandparenthood research with analyses of language use data using an intensive longitudinal design. The within-person links between positive word use and self-reported emotions were smaller than in previous research (Carlier et al., 2022). Rather than representing a methodological shortcoming, the modest association between self-reports and language indicators speaks for the inclusion of multiple methods to investigate psychological phenomena. Discrepancies between self-report and behavioral data are often reported in past research, potentially reflecting their distinct foci and the absence of shared method variance (Demiray et al., 2020; Sun et al., 2020; Vine et al., 2020). Yet, despite the limited within-person association of the two methods across all observations, we consistently found that positively valenced words were associated with grandchild contact across all comparison conditions. To our knowledge, this is the first study to employ multiple assessment methods in exploring the emotional impact of grandchild contact in daily life.

However, this study also has some limitations that might guide future research. First, the dataset lacks details on grandparent–grandchild contact, such as who initiated it, whether it was desired, and what it involved (e.g., play, emotional support, or physical care from grandchild to grandparent). This limits the interpretation of the associations. We also cannot determine the direction of effects, as grandparents’ emotions may influence when contact occurs, just as contact may shape emotions. The experiences of the grandparents in this sample might also differ. Although most participants had older grandchildren, 11.2% reported at baseline that they provided care to grandchildren, yet it is unknown whether such caregiving occurred during the study period. A common factor, however, is that all these grandparents previously identified their grandchildren as close social network members. Although this might have impacted their emotional reactions during contact, the effects of grandchild contact remained even when compared with interactions involving other close social partners.

Assessments also referred to the preceding 3 hr but lacked information on interaction duration or timing, potentially allowing emotions to return to baseline following interactions. Although the sample included more than 100 participants, with an average of 20 assessments per participant, it was too small to examine cross-level interactions and had limited power to detect between-person effects (see preregistered power analysis). Furthermore, to increase the sample size, some comparison groups overlapped (e.g., spouses were part of both the contact with others and the more restrictive alone group), which may have obscured certain effects.

Due to the small number of grandchild contact moments, we combined face-to-face and remote interactions. Future research should aim to compare the two forms of contact, as both are prevalent in maintaining contact with grandchildren (Chereches et al., 2025), but some emotions might be attenuated during remote contact (Grünjes et al., 2024). Lastly, we controlled for gender, but future research should examine gender differences in grandchild care, as caregiving roles may differ between grandmothers and grandfathers.

Finally, these findings should be interpreted in the context of this particular sample (U.S. American sample), and may not generalize to other populations or cultural contexts, where the meaning and consequences of grandparent–grandchild contact can differ (Chan et al., 2023).

## Conclusion

This study combined multiple assessment methods (EMA and EAR) to investigate whether contact with grandchildren affects grandparents’ emotions. Contact with grandchildren was consistently associated with more positive emotions (particularly love and pride) compared to time spent alone, with a spouse, or with others. We also found some (less robust) evidence linking grandchild contact to increased negative emotions. Grandparents also used more positive words when with grandchildren than in other contexts.

## Supplementary Material

gbag043_Supplementary_Data

## Data Availability

Data of this study are available at https://www.icpsr.umich.edu/web/NACDA/studies/38570. Preregistration and materials of this project are available on https://osf.io/9w5fx/?view_only=69f80f8832b34c16abe2e1dacbf48bbd.
