# Supplementary material for: Grandchild contact and grandparents’ positive and negative emotions in daily life: an analysis using self-report and language use data
Source: J Gerontol B Psychol Sci Soc Sci. 2026 Mar 21;81(5):gbag043. doi: 10.1093/geronb/gbag043 (PMC13143431; doi:10.1093/geronb/gbag043)
Supplement: gbag043_Supplementary_Data [file gbag043_supplementary_data.docx]

**Supplementary Material: Contact with Grandchildren and Grandparents’ Positive and Negative Emotions in Daily Life using Self-Report and Emotion Word Use Data**

**Contents**

[Description of the Sample and Study Variables 2](#_Toc223512455)

[Further Details on Analysis 4](#_Toc223512456)

[Adding Autoregressive Effects and Time of Assessment as Additional Within-Person Predictors 7](#_Toc223512457)

[Contextual Factors: The Effect of Pleasantness 10](#_Toc223512458)

[Adding Perceived Pain as a Within-Person Covariate 13](#_Toc223512459)

[Between-Person Findings of the Multilevel Models 16](#_Toc223512460)

# **Description of the Sample and Study Variables**

In the publicly available dataset, the age of participants was grouped in brackets. In the following table, we report the age distribution of the sample.

| Age group | 65-67 years | 68-70 years | 71-73 years | 74-76 years | 77-79 years | 80-82 years | 83-85 years | 86-88 years | 89 + years |
| --- | --- | --- | --- | --- | --- | --- | --- | --- | --- |
| Number participants | 22 | 16 | 24 | 21 | 11 | 15 | 12 | 4 | 1 |

**Table S1.** Descriptive Information on Background and Study Variables

|  | *M* | *SD* |
| --- | --- | --- |
| Age | 2.81 | 2.1 |
| Education | 5.88 | 1.53 |
| Gender (women) | 70% | - |
| Married | 55.2% | - |
| Number of Grandchildren | 5.5 | 5.32 |
| Subjective Health | 3.71 | 1.01 |
| Depression | 4.46 | 4.07 |
| Contact with Grandchildren | 0.25 | 0.43 |
| Pleasantness contact with grandchildren | 4.78 | 0.49 |
| Pleasantness contact with friends | 4.80 | 0.49 |
| Pleasantness contact with children | 4.63 | 0.64 |
| Pleasantness social contact (mean) | 4.65 | 0.58 |
| Frequency being Alone | 0.10 | 0.30 |
| Energetic | 3.07 | 1.05 |
| Nervous | 1.23 | 0.54 |
| Irritated | 1.22 | 0.56 |
| Proud | 2.60 | 1.36 |
| Bored | 1.13 | 0.43 |
| Lonely | 1.10 | 0.38 |
| Content | 3.84 | 0.95 |
| Sad | 1.17 | 0.47 |
| Loved | 4.05 | 1.06 |
| Calm | 3.97 | 0.86 |
| Negative emotions | 1.17 | 0.34 |
| Positive emotions | 3.50 | 0.73 |
| EAR positive emotions | 2.04 | 2.57 |
| EAR negative emotions | 0.28 | 0.64 |
| Age group youngest grandchild | 3.92 | 2.30 |

Note. Education was assessed as follows:1 = No Formal Education; 2 = Elementary School; 3 = Some High School; 4 = High School; 5 = Some College/Vocation or Trade School; 6 = College Graduate; 7 = Post College (no additional degree); 8 = Advanced degree; 9 = Other (albeit no participants chose this option); Subjective health: a higher score indicated better subjective health; Depression = Depression score, calculated over a sum of 11 items, where a higher score indicated more symptoms; Contact with Grandchildren = averaged contact with grandchildren over the study period; Pleasantness contact with grandchildren = Pleasantness interaction with grandchildren; Pleasantness contact with friends = Pleasantness interaction with friends; Pleasantness contact with children = Pleasantness interaction with children; Pleasantness social contact (mean) = Pleasantness interaction with all social members, averaged; Frequency being Alone = Averaged moments participants were alone; Negative emotion = Averaged negative emotions; Positive emotions = Averaged positive emotions; EAR positive emotion = Spoken Positive Emotions; EAR negative emotion = Spoken negative emotions; Age group youngest grandchild = Age of the youngest grandchild with whom contact took place during study period with group 3 marking grandchildren between the ages of 11 to 15 years and group 4 marking grandchildren between the ages of 16 to 20 years

# **Further Details on Analysis**

To test our hypotheses, we used multilevel structural equation modeling in Mplus, with the Mplus default diffuse priors in all models (Asparouhov & Muthén, 2010). We used the Bayes estimator and latent centering for our Level 1 predictors. Predictors were split in between- and within-person latent components, where the between-person components are similar to person means corrected for measurement error and the within-person components are time-specific deviations from these latent intercepts. We used random slopes for the focal predictors, as we expected the effects to differ between individuals. The estimated model was as follows:

Within-person:

Well-being*_ti_* = $\beta_{0i}$ + $\beta_{1i}$ ${\mathrm{Grandchild} \mathrm{Interaction}}_{ti}^{w}$ + $e_{ti}$

Between-person:

$\beta_{0i}$= $\gamma_{00}$+$\gamma_{01}{\mathrm{Grandchild} \mathrm{Interaction}}_{i}^{b}$ + $\gamma_{02}\mathrm{Women}_{i}$ + $\gamma_{03}\mathrm{Age}_{i}$ +$\gamma_{04}\mathrm{Married}_{i}$ +$u_{01}$

$\beta_{1i}$= $\gamma_{10}$+$u_{1i}$

On the within-person level, affective well-being (each emotion individually and grouped based on their valence) at time-point *t* for person *i* is equal to a person specific intercept $\beta_{0i}$, plus the person specific effect of social interactions $\beta_{1i}$, and a time point specific residual $e_{ti}$, assumed to be normally distributed with constant variance $e_{ti}$ ~ *N*(0, $\sigma^{2}$). At the between-person level, $\gamma_{00}$ represents the sample average for the well-being outcome, $u_{0i}$ is the deviation of a participant score on well-being from the sample average, $\gamma_{01}$ represents the between-person effect of social interaction, $\gamma_{10}$ represents the within-person effect of grandchild contact on momentary well-being (or the average slope), and $u_{1i}$ is the deviation of a participant from this sample average. Further, $\gamma_{02}$ represents the effect of gender on well-being, $\gamma_{03}$ is the effect of grandparents’ age on well-being and $\gamma_{04}$ represents the effect of marital status on well-being (where gender, age and marital status are control variables). We were specifically interested in the within-person effect of grandchild contact, $\gamma_{10}$which indicates if grandchild contact is related to momentary changes in well-being, yet we also report the between-person effect of grandchild contact, or the effect of $\gamma_{01}$. To make inferences, we used Bayesian credible intervals. We used the Potential Scale Reduction (PSR) criterion to assess model convergence, taking as convergences when factors were close to 1.01 (Brooks & Gelman, 1998). If values went above 1.01, we increased the number of iterations from 20000 to 40000.

**Table S2**. Correlations Between Background and Study Variables

|  | **Cont g** | **Pl. g** | **Pleas. f** | **Pleas. ch** | **Pleas. mean** | **Alone** | **Neg emotion** | **Pos emotions** | **EAR pos em** | **EAR neg em** |
| --- | --- | --- | --- | --- | --- | --- | --- | --- | --- | --- |
| Age | -0.06 | -0.08 | 0.08 | -0.01 | 0.08 | -0.03 | -0.02 | -0.15 | -0.20* | -0.16 |
| Edu | -0.36*** | -0.06 | 0.06 | -0.03 | 0.00 | 0.22* | -0.07 | -0.05 | 0.03 | -0.12 |
| Gender (women) | -0.03 | 0.07 | 0.19 | 0.05 | 0.03 | 0.16 | -0.08 | -0.02 | -0.14 | -0.12 |
| Married | -0.07 | 0.20* | 0.28* | 0.13 | 0.08 | 0.23* | -0.02 | 0.04 | 0.06 | -0.12 |
| No Grand | 0.20* | -0.02 | -0.60*** | -0.05 | -0.17 | -0.11 | 0.03 | -0.06 | -0.24** | 0 |
| Health | -0.25** | 0.27** | 0.04 | 0.20* | 0.24** | 0.17 | -0.37*** | 0.24** | 0.12 | -0.04 |
| Depression | 0.26** | -0.09 | -0.23 | -0.34*** | -0.31*** | -0.11 | 0.49*** | -0.32*** | -0.03 | 0.01 |
| Cont g | 1*** | -0.28** | -0.08 | -0.14 | -0.16 | -0.51*** | 0.29** | -0.26** | -0.08 | 0.21* |
| Pleas. g | -0.28** | 1*** | 0.17 | 0.48*** | 0.44*** | 0.11 | -0.22* | 0.34*** | 0.07 | -0.01 |
| Pl. f | -0.08 | 0.17 | 1*** | 0.33* | 0.69*** | 0.03 | -0.16 | 0.02 | 0.01 | -0.10 |
| Pl. ch | -0.14 | 0.48*** | 0.33* | 1*** | 0.91*** | 0.03 | -0.30** | 0.40*** | -0.28** | -0.01 |
| Pl. mean | -0.16 | 0.44*** | 0.69*** | 0.91*** | 1*** | 0.03 | -0.26** | 0.29*** | -0.23* | 0.02 |
| Alone | -0.51*** | 0.11 | 0.03 | 0.03 | 0.03 | 1*** | -0.14 | -0.02 | 0 | -0.21* |
| Energetic | -0.09 | 0.27** | 0.11 | 0.26** | 0.25** | -0.07 | -0.23** | 0.56*** | 0.11 | 0.04 |
| Nervous | 0.18* | -0.15 | -0.15 | -0.28** | -0.26** | -0.14 | 0.90*** | -0.34*** | -0.04 | 0.22* |
| Irritated | 0.20* | -0.23* | -0.19 | -0.30** | -0.33*** | -0.13 | 0.78*** | -0.31*** | 0.05 | 0.31*** |
| Proud | -0.05 | 0.27** | -0.04 | 0.25** | 0.19* | -0.13 | -0.09 | 0.72*** | 0.03 | 0.12 |
| Bored | 0.37*** | -0.11 | -0.20 | -0.18 | -0.09 | -0.12 | 0.79*** | -0.32*** | -0.08 | 0.32*** |
| Lonely | 0.17 | -0.11 | -0.01 | -0.17 | -0.15 | 0.03 | 0.83*** | -0.34*** | -0.14 | 0.14 |
| Content | -0.32*** | 0.27** | 0.06 | 0.33*** | 0.26** | 0.14 | -0.54*** | 0.78*** | 0.03 | -0.12 |
| Sad | 0.19* | -0.32*** | -0.07 | -0.31*** | -0.26** | -0.15 | 0.82*** | -0.32*** | -0.02 | 0.24** |
| Loved | -0.22* | 0.29** | 0 | 0.37*** | 0.22* | -0.06 | -0.25** | 0.79*** | 0.08 | 0 |
| Calm | -0.28** | 0.17 | 0.08 | 0.31*** | 0.25** | 0.09 | -0.49*** | 0.78*** | 0.01 | -0.13 |
| Neg emotions | 0.29** | -0.22* | -0.16 | -0.30** | -0.26** | -0.14 | 1*** | -0.40*** | -0.05 | 0.30** |
| Pos emotions | -0.26** | 0.34*** | 0.02 | 0.40*** | 0.29*** | -0.02 | -0.40*** | 1*** | 0.05 | -0.01 |
| EAR pos em | -0.08 | 0.07 | 0.01 | -0.28** | -0.23* | 0 | -0.05 | 0.05 | 1*** | 0.37*** |
| EAR neg em | 0.21* | -0.01 | -0.10 | -0.01 | 0.02 | -0.21* | 0.30** | -0.01 | 0.37*** | 1*** |
| Age young g | 0.22* | -0.14 | -0.24 | -0.19* | -0.08 | -0.12 | 0.21* | -0.16 | -0.05 | -0.04 |

Note. Education was assessed as follows:1 = No Formal Education; 2 = Elementary School; 3 = Some High School; 4 = High School; 5 = Some College/Vocation or Trade School; 6 = College Graduate; 7 = Post College (no additional degree); 8 = Advanced degree; 9 = Other (albeit no participants chose this option); No Grand = Total number of grandchildren; Health = Subjective health, where a higher score indicated better subjective health; Depression = Depression score, calculated over a sum of 11 items, where a higher score indicated more symptoms; see codebook descriptions in Fingerman et al., 2022); Cont g = averaged contact with grandchildren over the study period; Pl/Pleas g = pleasantness interaction with grandchildren; Pl/Pleas f = Pleasantness interaction with friends; Pl/Pleas ch = Pleasantness interaction with children; Pl/Pleas mean = Pleasantness interaction with all social members, averaged; Alone = Averaged moments participants were alone; Neg emotion = Averaged negative emotions; Pos emotions = Averaged positive emotions; EAR pos em = Spoken Positive Emotions; EAR neg em = Spoken negative emotions; Age young g = Age of the youngest grandchild with whom contact took place during study period.

# **Adding Autoregressive Effects and Time of Assessment as Additional Within-Person Predictors**

Since prior emotions may linger and influence subsequent emotions, we included levels of the previous emotion as a predictor of current emotion levels at the within-person level. These analyses were limited to the dataset that included all available assessment points (i.e., both grandchild-contact and no-contact occasions), which retained the time-series structure necessary for modeling temporal dependencies.

We first estimated models that included only the autoregressive control (i.e., prior emotion predicting current emotion). In follow-up models, we additionally included time in study as a covariate, coding it so that each participant’s first assessment was 0 and subsequent assessments increased incrementally according to their position in the study.

After including the previous emotion as a within-person covariate, all previously observed effects remained significant, except for the association between grandchild contact and negative emotions, which was no longer significant. Adding time in study as a further predictor yielded a comparable pattern of results (see Tables S2 to S6).

**Table S3.** *Unstandardized and (Standardized) Within-Person Effect of Previous Emotion Predicting Current Emotion*

|  | **Estimate** | **95% CI** |
| --- | --- | --- |
| Energetic | **0.269 (.233)** | **[0.223, 0.315]** |
| Content | **0.133 (.118)** | **[0.086, 0.181]** |
| Loved | **0.193 (.151)** | **[0.150, 0.237]** |
| Proud | **0.188 (.156)** | **[0.142, 0.234]** |
| Calm | **0.105 (.090)** | **[0.057, 0.153]** |
| Bored | 0.012 (.006) | [-0.038, 0.061] |
| Lonely | **0.209 (.080)** | **[0.157, 0.262]** |
| Sad | **0.080 (.045)** | **[0.029, 0.130]** |
| Irritated | **0.217 (.147)** | **[0.167, 0.267]** |
| Nervous/Worried | **0.212 (.134)** | **[0.164, 0.259]** |
| Negative Emotions | **0.286 (.190)** | **[0.235, 0.336]** |
| Positive Emotions | **0.249 (.232)** | **[0.205, 0.292]** |

Note. Note that the analysis is based on the whole sample (grandchildren present versus grandchild contact absent). Shown are unstandardized estimates with the corresponding 95% credible intervals, while the standardized estimates are displayed in parentheses. Following the recommendations of de Haan-Rietdijk et al. (2016), when creating the lagged emotions, we did not lag the first observation of each day, such that we do not predict morning emotion by the previous last assessment, which occurred in the evening. Hence, these analyses were based on less observations (1799).

**Table S4.** *Unstandardized and (Standardized) Within-Person Effect of Time from Models Controlling for Previous Self-Reported Emotion*

|  | **Estimate** | **95% CI** |
| --- | --- | --- |
| Energetic | -0.002 (-.016) | [-0.007, 0.003] |
| Content | -0.003 (-.030) | [-0.008, 0.001] |
| Loved | -0.001 (-.008) | [-0.005, 0.003] |
| **Proud** | **0.014 (.125)** | **[0.010, 0.019]** |
| **Calm** | **-0.009 (-.085)** | **[-0.013, -0.004]** |
| Bored | -0.001 (-.027) | [-0.004, 0.001] |
| Lonely | 0.002 (.037) | [0.000, 0.005] |
| Sad | 0.001 (.021) | [-0.001, 0.004] |
| Irritated | 0.000 (.004) | [-0.003, 0.004] |
| Nervous/Worried | 0.002 (.034) | [-0.001, 0.005] |
| Negative Emotions | 0.001 (.004) | [-0.001, 0.003] |
| Positive Emotions | 0.000 (-.008) | [-0.003, 0.002] |

Note. Note that the analysis is based on the whole sample (grandchildren present versus grandchild contact absent). Shown are unstandardized estimates with the corresponding 95% credible intervals, while the standardized estimates are displayed in parentheses. Following the recommendations of de Haan-Rietdijk et al. (2016), when creating the lagged emotions, we did not lag the first observation of each day, such that we do not predict morning emotion by the previous last assessment, which occurred in the evening. Hence, these analyses were based on less observations (1799).

**Table S5.** *Explained Variance in Models without controlling for Previous Emotion and Time/ Controlling for Previous Emotion Level/Controlling for Previous Emotion Level and Time*

|  | **Model Explained Variance** | **Including Previous Emotion Level: Explained Variance [95% CI]** | **Including Previous Emotion Level and Time: Explained Variance [95% CI]** |
| --- | --- | --- | --- |
| Energetic | 0.006 [0.001, 0.016] | 0.071 [0.052, 0.095] | 0.073 [0.052, 0.097] |
| Content | 0.031 [0.014, 0.052] | 0.047 [0.027, 0.074] | 0.046 [0.025, 0.071] |
| Loved | 0.033 [0.018, 0.050] | 0.060 [0.039, 0.084] | 0.061 [0.041, 0.082] |
| Proud | 0.031 [0.017, 0.048] | 0.060 [0.041, 0.082] | 0.075 [0.053, 0.101] |
| Calm | 0.015 [0.003, 0.033] | 0.021 [0.009, 0.038] | 0.027 [0.014, 0.046] |
| Bored | 0.042 [0.026, 0.062] | 0.055 [0.035, 0.078] | 0.058 [0.036, 0.083] |
| Lonely | 0.028 [0.012, 0.052] | 0.041 [0.027, 0.059] | 0.040 [0.026, 0.057] |
| Sad | 0.080 [0.058, 0.105] | 0.111 [0.087, 0.136] | 0.111 [0.087, 0.137] |
| Irritated | 0.007 [0.001, 0.024] | 0.050 [0.032, 0.072] | 0.049 [0.031, 0.073] |
| Nervous/Worried | 0.039 [0.019, 0.059] | 0.054 [0.036, 0.080] | 0.054 [0.036, 0.077] |
| Negative Emotions | 0.070 [0.048, 0.093] | 0.106 [0.078, 0.136] | 0.108 [0.076, 0.139] |
| Positive Emotions | 0.024 [0.012, 0.041] | 0.078 [0.057, 0.104] | 0.080 [0.058, 0.104] |

Note. Results come from separate models. Column “Model Explained Variance pertains models with no additional within-person predictors than contact with grandchildren”. Column “Including Previous Emotion Level: Explained Variance and 95% CI” pertains to models with previous emotion level as an additional within-person predictor; Column “Including Previous Emotion Level and Time: Explained Variance and 95% CI” pertains to models with previous emotion level and time as additional within-person predictors.

**Table S6.** *Model Results: Unstandardized and (Standardized) Within-Person Findings Controlling for Previous Self-Reported Emotion and Time in the Study*

|  | **Estimate** | **95% CI** |
| --- | --- | --- |
| Energetic | 0.050 (.024) | [-0.047, 0.142] |
| Content | 0.035 (.019) | [-0.066, 0.134] |
| **Loved** | **0.112 (.074)** | **[0.038, 0.190]** |
| **Proud** | **0.115 (.068)** | **[0.026, 0.201]** |
| Calm | -0.001 (-.003) | [-0.073, 0.083] |
| Bored | 0.030 (.028) | [-0.024, 0.088] |
| Lonely | 0.005 (.007) | [-0.038, 0.052] |
| Sad | 0.082 (.045) | [-0.023, 0.190] |
| Irritated | -0.019 (-.015) | [-0.082, 0.045] |
| Nervous/Worried | 0.042 (.036) | [-0.014, 0.101] |
| Negative Emotions | 0.030 (.043) | [-0.010, 0.075] |
| **Positive Emotions** | **0.066 (.068)** | **[0.018, 0.114]** |

Note. Note that the analysis is based on the whole sample (grandchildren present versus grandchild contact absent). Shown are unstandardized estimates with the corresponding 95% credible intervals, while the standardized estimates are displayed in parentheses. Following the recommendations of de Haan-Rietdijk et al. (2016), when creating the lagged emotions, we did not lag the first observation of each day, such that we do not predict morning emotion by the previous last assessment, which occurred in the evening. Hence, these analyses were based on less observations (1799).

# **Contextual Factors: The Effect of Pleasantness**

We first compared the pleasantness of different types of social contact. Pleasantness was assessed after each reported social interaction. For every individual from ones’ close social network with whom participants interacted, they rated the question *“How pleasant was this interaction for you?”* on a scale from 1 (*unpleasant*), 2 (*a little unpleasant*), 3 (*neutral*), 4 (*a little pleasant*), to 5 (*pleasant*).

For the initial descriptive comparisons, we averaged pleasantness ratings across the study period separately for moments when contact with grandchildren was reported and for other types of close social interactions. Dependent *t*-tests were used to compare these average pleasantness scores across contact types (see results in Table S2)

In the multilevel models, we aggregated all pleasantness ratings reported at each assessment point to create a within-person covariate representing the mean pleasantness of interactions at that occasion. These analyses were conducted only on observations when social interactions occurred. Pleasantness was modeled at both the within-person and between-person levels to capture momentary fluctuations as well as stable individual differences.

**Table S7.** *Comparing Pleasantness of Grandchild Contact to Contacts with Other Social Groups*

| **Grandchild contact compared to contact with** | ***t*** | ***df*** | ***p*** | **Cohen’s d** |
| --- | --- | --- | --- | --- |
| Others (overall) | 2.08 | 115 | .039 | 0.19 |
| Spouse | 1.01 | 62 | .318 | 0.13 |
| Children | 2.10 | 106 | .038 | 0.20 |
| Friends | -0.31 | 48 | .761 | -0.04 |

Note. Spouse reflects contact with spouse versus contact with grandchildren. Children reflects contact with children versus contact with grandchildren. Friends reflects contact with friends versus contact with grandchildren. The degrees of freedom differ, as these results come from different subsamples (not all grandparents had contact with their friends over study period/or reported them as close social members to be able to identify them). Note that we averaged all pleasantness ratings per individual over the study period.

**Table S8.** *Within-Person Findings: Unstandardized (and Standardized) Estimates Comparing Grandchild Contact to Contact with Close Others while Controlling for Level 1 Pleasantness*

|  | **Grandchildren not present** | |
| --- | --- | --- |
|  | **Estimate** | **95% CI** |
| Energetic | 0.025 (.008) | [-0.065, 0.118] |
| Content | 0.065 (.036) | [-0.031, 0.155] |
| Loved | **0.070 (.056)** | **[0.004, 0.139]** |
| Proud | **0.111 (.064)** | **[0.011, 0.210]** |
| Calm | -0.012 (-.004) | [-0.089, 0.067] |
| Bored | 0.025 (.009) | [-0.039, 0.092] |
| Lonely | 0.022 (.028) | [-0.020, 0.066] |
| Sad | 0.076 (.061) | [-0.003, 0.156] |
| Irritated | -0.004 (-.002) | [-0.063, 0.056] |
| Nervous/Worried | 0.047 (.039) | [-0.016, 0.113] |
| Negative Emotions | 0.037 (.052) | [-0.004, 0.082] |
| Positive Emotions | **0.060 (.059)** | **[0.008, 0.113]** |
| EAR Negative Emotions | -0.023 (-.004) | [-0.113, 0.071] |
| EAR Positive Emotions | **0.546 (.094)** | **[0.165, 0.908]** |

Note. Shown are unstandardized estimates, and in parentheses are the standardized estimates. In the squared brackets, we present 95% credible intervals. Coefficients in bold mark effects for which the credibility interval does not include 0. Grandchildren not present = results on the full sample, comparing situations of grandchild contact with interacting with other social members or time spent alone or with the partner only; EAR Positive Emotions = Use of positive emotion-related words; EAR Negative = Use of negative emotion-related words.

**Table S9.** *Within-Person Explained Variance in Models Comparing Grandchild Contact to Contact with Others as compared to Models including Pleasantness of Social Interactions at Level 1 as Additional Covariate*

|  | **Original Model Explained Variance [95% CI]** | **Including Pleasantness Explained Variance [95% CI]** |
| --- | --- | --- |
| Energetic | 0.008 [0.001, 0.018] | 0.006 [0.001, 0.021] |
| Content | 0.036 [0.018, 0.061] | 0.043 [0.024, 0.068] |
| Loved | 0.009 [0.001, 0.023] | 0.015 [0.005, 0.029] |
| Proud | 0.037 [0.020, 0.055] | 0.042 [0.024, 0.063] |
| Calm | 0.011 [0.002, 0.030] | 0.026 [0.010, 0.047] |
| Bored | 0.075 [0.051, 0.098] | 0.082 [0.055, 0.106] |
| Lonely | 0.020 [0.006, 0.042] | 0.022 [0.005, 0.046] |
| Sad | 0.092 [0.067, 0.117] | 0.140 [0.111, 0.171] |
| Irritated | 0.012 [0.002, 0.033] | 0.051 [0.030, 0.074] |
| Nervous/Worried | 0.040 [0.020, 0.065] | 0.066 [0.040, 0.094] |
| Negative Emotions | 0.075 [0.050, 0.102] | 0.139 [0.107, 0.176] |
| Positive Emotions | 0.029 [0.013, 0.048] | 0.034 [0.018, 0.057] |
| EAR Negative Emotions | 0.051 [0.030, 0.076] | 0.050 [0.029, 0.078] |
| EAR Positive Emotions | 0.162 [0.126, 0.200] | 0.163 [0.129, 0.202] |

Note. EAR Positive Emotions = Use of positive emotion-related words; EAR Negative = Use of negative emotion-related words.

**Table S10.** *Within-Person Findings: Estimates for the Effect of Pleasantness of Social Interactions on Each Emotion*

|  | **Effect of Pleasantness** | |
| --- | --- | --- |
|  | **Estimate** | **95% CI** |
| Energetic | -0.030 (-.023) | [-0.100, 0.037] |
| Content | **0.125 (.107)** | **[0.065, 0.183]** |
| Loved | **0.061 (.062)** | **[0.010, 0.111]** |
| Proud | **0.069 (.059)** | **[0.010, 0.128]** |
| Calm | **0.116 (.111)** | **[0.061, 0.171]** |
| Bored | **-0.039 (-.073)** | **[-0.067, -0.011]** |
| Lonely | -0.024 (-.044) | [-0.053, 0.006] |
| Sad | **-0.156 (-.231)** | **[-0.188, -0.124]** |
| Irritated | **-0.160 (-.193)** | **[-0.203, -0.116]** |
| Nervous/Worried | **-0.127 (-.173)** | **[-0.165, -0.088]** |
| Negative Emotions | **-0.111 (-.277)** | **[-0.131, -0.090]** |
| Positive Emotions | **0.063 (.095)** | **[0.029, 0.096]** |
| EAR Negative Emotions | -0.002 (-.003) | [-0.067, 0.064] |
| EAR Positive Emotions | -0.006 (-.001) | [-0.242, 0.234] |

Note. EAR Positive Emotions = Use of positive emotion-related words; EAR Negative = Use of negative emotion-related words.

# **Adding Perceived Pain as a Within-Person Covariate**

We run a series of supplementary analyses to see if the effects observed within-person are potentially driven by grandparents’ perceived pain. Pain was assessed in daily life with a single item: *“How much pain or discomfort did you experience in the past 3 hours?”. Responses were given on a 4-point scale ranging from 0 (no pain) to 3 (severe pain).* The variable was latent-mean centered and entered both at Level-1 (within-person) and at Level-2 as a predictor for emotions in the models, including all observations (i.e., comparing contact with grandchildren with the absence of contact with grandchildren).

We started by examining whether moments of grandchild contact co-occurred with higher levels of pain. The within-person correlation between grandchild contact and pain was not significant (*r* = .01, *p* = .513), suggesting that grandchild contact did not systematically coincide with changes in pain levels. Pain was related to within-person emotions in the expected direction—positively with negative emotions (*r* = .12, *p* < .001) and negatively with positive emotions (*r* = –.12, *p* < .001). Given that pain has been added as a Level-1 covariate, the well-being outcomes are corrected for fluctuations in pain. As can be seen from Table S6, most of the effects were unchanged compared to the original results and pain emerged as a significant within-person predictor for emotions (see Table S7).

**Table S11.** *Unstandardized and (Standardized) Within-Person Effect Controlling for Perceived Pain*

|  | **Estimate** | **95% CI** |
| --- | --- | --- |
| Energetic | 0.066 (.026) | [-0.017, 0.150] |
| Content | 0.055 (.031) | [-0.038, 0.147] |
| Loved | **0.142 (.089)** | **[0.063, 0.219]** |
| Proud | **0.151 (.084)** | **[0.060, 0.243]** |
| Calm | -0.020 (-.014) | [-0.093, 0.053] |
| Bored | 0.020 (.015) | [-0.031, 0.076] |
| Lonely | 0.029 (.034) | [-0.015, 0.075] |
| Sad | **0.084 (.064)** | **[0.005, 0.163]** |
| Irritated | 0.000 (.002) | [-0.054, 0.056] |
| Nervous/Worried | **0.062 (.053)** | **[0.002, 0.125]** |
| Negative Emotions | **0.046 (.060)** | **[0.003, 0.092]** |
| Positive Emotions | **0.089 (.084)** | **[0.038, 0.138]** |
| EAR Negative Emotions | 0.008 (-.003) | [-0.075, 0.091] |
| EAR Positive Emotions | **0.525 (.081)** | **[0.173, 0.871]** |

Note. Note that the analysis is based on the whole sample (grandchildren present versus grandchild contact absent). Shown are unstandardized estimates with the corresponding 95% credible intervals, while the standardized estimates are displayed in parentheses.

**Table S12.** *Unstandardized and (Standardized) Within-Person Effect of Perceived Pain*

|  | **Estimate** | **95% CI** |
| --- | --- | --- |
| Energetic | **-0.234 (-.127)** | **[-0.306, -0.161]** |
| Content | **-0.125 (-.081)** | **[-0.185, -0.064]** |
| Loved | -0.018 (-.014) | [-0.073, 0.036] |
| Proud | 0.007 (.004) | [-0.056, 0.071] |
| Calm | **-0.171 (-.122)** | **[-0.228, -0.116]** |
| Bored | **0.041 (.055)** | **[0.011, 0.069]** |
| Lonely | **0.067 (.089)** | **[0.037, 0.096]** |
| Sad | **0.088 (.094)** | **[0.052, 0.124]** |
| Irritated | **0.116 (.104)** | **[0.072, 0.160]** |
| Nervous/Worried | **0.068 (.068)** | **[0.029, 0.107]** |
| Negative Emotions | **0.075 (.134)** | **[0.054, 0.097]** |
| Positive Emotions | **-0.111 (-.119)** | **[-0.148, -0.074]** |
| EAR Negative Emotions | **0.050 (.033)** | **[-0.024, 0.122]** |
| EAR Positive Emotions | -0.186 (-.034) | [-0.468, 0.101] |

Note. Note that the analysis is based on the whole sample (grandchildren present versus grandchild contact absent). Shown are unstandardized estimates with the corresponding 95% credible intervals, while the standardized estimates are displayed in parentheses.

**Table S13.** *Within-Person Explained Variance in Models Comparing Grandchild Contact to No Grandchild Contact as Compared to Models including Pain at Level 1 as an Additional Covariate*

|  | **Original Model Explained Variance [95% CI]** | **Including Pain Explained Variance [95% CI]** |
| --- | --- | --- |
| Energetic | 0.006 [0.001, 0.016] | 0.022 [0.012, 0.036] |
| Content | 0.031 [0.014, 0.052] | 0.038 [0.012, 0.059] |
| Loved | 0.033 [0.018, 0.050] | 0.034 [0.021, 0.050] |
| Proud | 0.031 [0.017, 0.048] | 0.030 [0.015, 0.048] |
| Calm | 0.015 [0.003, 0.033] | 0.032 [0.016, 0.052] |
| Bored | 0.042 [0.026, 0.062] | 0.048 [0.029, 0.068] |
| Lonely | 0.028 [0.012, 0.052] | 0.037 [0.020, 0.057] |
| Sad | 0.080 [0.058, 0.105] | 0.092 [0.071, 0.113] |
| Irritated | 0.007 [0.001, 0.024] | 0.018 [0.008, 0.036] |
| Nervous/Worried | 0.039 [0.019, 0.059] | 0.043 [0.023, 0.064] |
| Negative Emotions | 0.070 [0.048, 0.093] | 0.086 [0.065, 0.110] |
| Positive Emotions | 0.024 [0.012, 0.041] | 0.039 [0.024, 0.059] |
| EAR Negative Emotions | 0.043 [0.026, 0.066] | 0.046 [0.028, 0.068] |
| EAR Positive Emotions | 0.142 [0.112, 0.172] | 0.144 [0.114, 0.176] |

Note. EAR Positive Emotions = Use of positive emotion-related words; EAR Negative = Use of negative emotion-related words.

# **Between-Person Findings of the Multilevel Models**

Although it was not the main focus of this study, we examined whether grandparents with more grandchild contact during the study period differed in their emotions and word use compared to those with less contact (see Table 4 for results). We analyzed this in the full observations sample.

**Self-Reported Emotions**

Based on all observations, we found that grandparents who had more contact with their grandchildren during the study period, generally experienced less positive affect (i.e., content, loved, calm) and more negative affect (i.e., bored, irritated, nervous/worried) compared to those grandparents who had less contact with their grandchildren during that time.

**Spoken Words**

Grandparents with more grandchild contact used more negatively-valenced words compared to those with less contact. Positive spoken words did not differ between grandparents with more or less grandchild contact.

**Table S14.** *Between-Person Findings: Unstandardized and (Standardized) Estimates of the Full Sample*

|  | **Estimate** | **95% CI** |
| --- | --- | --- |
| Energetic | -0.308 (-.091) | [-0.988, 0.351] |
| Content | **-1.145 (-.329)** | **[-1.812, -0.483]** |
| Loved | **-0.984 (-.228)** | **[-1.785, -0.199]** |
| Proud | -0.473 (-.083) | [-1.570, 0.644] |
| Calm | **-0.886 (-.278)** | **[-1.494, -0.290]** |
| Bored | **0.394 (.221)** | **[0.070, 0.767]** |
| Lonely | 0.111 (.105) | [-0.106, 0.330] |
| Sad | 0.215 (.174) | [-0.044, 0.479] |
| Irritated | **0.374 (.250)** | **[0.076, 0.675]** |
| Nervous/Worried | 0.339 (.192) | [-0.007, 0.688] |
| Negative emotions | **0.352 (.274)** | **[0.110, 0.599]** |
| Positive emotions | **-0.753 (-.251)** | **[-1.324, -0.180]** |
| EAR Negative Em | **0.322 (.312)** | **[0.055, 0.578]** |
| EAR Positive Em | -0.895 (-.163) | [-2.049, 0.288] |

Note. Shown are unstandardized estimates with standardized estimates in parentheses. In the squared brackets, we present 95% credible intervals. Coefficients in bold mark effects for which the credibility interval does not include 0. EAR Positive Em = Spoken positive emotion-related words; EAR Negative Em = Spoken negative emotion-related words.

**References**

Asparouhov, T., & Muthén, B. (2010, September). *Bayesian analysis of latent variable models using Mplus*. <https://www.statmodel.com/download/BayesAdvantages18.pdf>

Brooks, S. P., & Gelman, A. (1998). General methods for monitoring convergence of iterative simulations. *Journal of Computational and Graphical Statistics*, 7(4), 434–455. <https://doi.org/10.1080/10618600.1998.10474787>

de Haan-Rietdijk, S., Kuppens, P., & Hamaker, E. L. (2016). What's in a day? A guide to decomposing the variance in intensive longitudinal data. *Frontiers in Psychology*, *7*, 891. <https://doi.org/10.3389/fpsyg.2016.00891>

Fingerman, Karen L., Charles, Susan T., and Birditt, Kira S. Daily Experiences and Well-being in Late Life, Austin, Texas, 2016-2017. Inter-university Consortium for Political and Social Research [distributor], 2022-11-28. <https://doi.org/10.3886/ICPSR38570.v1>
